# Effects of ashwagandha (*Withania somnifera*) root extract on aging‐related changes in healthy geriatric dogs: A randomized, double‐blinded placebo‐controlled study

**DOI:** 10.1002/vms3.1556

**Published:** 2024-07-30

**Authors:** Kala Kumar Bharani, Ashok Kumar Devarasetti, Latha Carey, Amit Khurana, Rajesh Kollipaka, Donga Durga Veera Hanuman, Vinaya Sree Chetla, Anil Kumar Banothu

**Affiliations:** ^1^ Department of Veterinary Pharmacology and Toxicology College of Veterinary Science (CVSc) PVNRTVU Hyderabad Telangana India; ^2^ Department of Veterinary Biochemistry College of Veterinary Science (CVSc) PVNRTVU Warangal Telangana India; ^3^ Department of Veterinary Surgery & Radiology College of Veterinary Science (CVSc) PVNRTVU Warangal Telangana India; ^4^ Department of Veterinary Pharmacology and Toxicology College of Veterinary Science (CVSc), PVNRTVU Hyderabad Telangana India; ^5^ Cure Pet Clinic Hanamkonda Telangana India; ^6^ Department of Veterinary Physiology College of Veterinary Science (CVSc) PVNRTVU Hyderabad Telangana India

**Keywords:** adaptogen, aging‐related changes, ashwagandha root extract, geriatric dogs, stress

## Abstract

**Background and aim:**

This study aimed to explore the clinical potential of *Withania somnifera*/ashwagandha root extract (ARE) to mitigate age‐related changes in healthy geriatric dogs. We hypothesized that ARE can reduce the effects of advancing age, including physiological changes, immune response decline and susceptibility to diseases, by its immunomodulatory effects.

**Methods:**

A randomized, double‐blind, placebo‐controlled trial was conducted in Telangana, India, from July 2022 to September 2022. Twenty apparently healthy dogs, aged 8 years or older, were enrolled. The dogs were divided into two groups to receive ARE (15 mg/kg, once daily, orally) or a placebo control. Various parameters, including serum cortisol levels, haematological profiles, biochemical markers, antioxidant indicators and anti‐inflammatory responses, were assessed at the initiation of study, day 30, and day 60.

**Results:**

The erythrocyte count and haemoglobin levels were significantly increased with ARE (*p *< 0.001), whereas leukocyte count decreased (*p *< 0.05). Moreover, significant decreases in important markers of liver function (alanine aminotransferase, aspartate aminotransferase, albumin and globulin; *p *< 0.001 at day 60), as well as kidney function markers (creatinine and blood urea nitrogen; *p *< 0.001 at days 30 and 60), were observed in ARE‐treated dogs compared to the placebo control group. In addition, the levels of markers of oxidative stress (superoxide dismutase, catalase, glutathione and malondialdehyde) were significantly modulated by ARE intervention, indicating strong antioxidant effects. Interestingly, serum cortisol levels reduced significantly with ARE (*p *< 0.001). Compared to baseline, ARE significantly decreased key inflammatory markers, including interferon‐γ, tumour necrosis factor‐α, nuclear factor kappa light chain enhancer of activated B cells and interleukin‐10 (*p *< 0.001) levels at day 60.

**Conclusion:**

In conclusion, the findings of this study suggest that ARE has adaptogenic properties in healthy geriatric dogs by improving haematological and biochemical profiles, enhancing antioxidant defence, reducing stress and modulating inflammatory responses.

## Graphical abstract

The schematic provides the overview of the study design and the methodology that was employed to assess the pharmacological effects of ARE in obese geriatric dogs. Important clinical and biochemical markers were investigated to ascertain the efficacy of ARE.

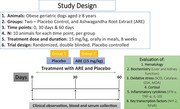

## 1 INTRODUCTION

Over the past 100 years, humans have achieved a significant increase in longevity, especially in developed nations (Hoffman et al., 2018; Sándor & Kubinyi, 2019). Similarly, substantial improvements have been witnessed in health, hygienic, nutritional and medical care for dogs during the last few decades, contributing to a significant increase in their longevity (Chapagain et al., 2018).

Aging is a physiological process with differential changes in a variety of cellular and biological markers (Simpson et al., 2017). Geriatric age is a critical phase that needs specialized attention for nutrition and mobility. Geriatric age induces a wide range of biological alterations including modified behavioural, structural and functional patterns that signal a general decline in the overall wellbeing (Pati et al., 2015). The unfavourable alterations that occur in geriatric age are characterized by a reduced ability to adapt to stress, homeostatic imbalance and elevated risk of diseases that may lead to death. Collectively, these changes are referred to as senescence and must be recognized early to initiate interventions to slow or reduce the influence on the animal's quality of life. Mechanistically, senescence is characterized by the elevation of an inflammatory response and increased oxidative damage, which manifest as a low‐level chronic proinflammatory state (Alexander et al., 2018).

The interplay of oxidative stress and inflammation is crucial in aging. The levels of physiological antioxidant defence enzymes like superoxide dismutase (SOD), catalase and glutathione (GSH) tend to reduce during the process of aging (Alexander et al., 2018). Further, increased cellular oxidative stress also culminates in the form of enhanced lipid peroxidation markers like malondialdehyde (MDA) (Todorova et al., 2005). On the other side, the inflammatory state is characterized by an overall increase in cytokines like interferon‐γ (IFN‐γ), tumour necrosis factor‐α (TNF‐α) and interleukin‐10 (IL‐10) (Day, 2010). Their levels are regulated by master transcription factors like nuclear factor erythroid 2‐related factor 2 (Nrf‐2) and nuclear factor kappa light chain enhancer of activated B cells (NFκB) (Ahmed et al., 2017; Biswas & Bagchi, 2016).

As with humans, dogs also experience a plethora of changes during aging, including loss of general robustness and resilience due to loss of muscle mass (Arbeev et al., 2016). This dramatically impacts the morbidity and longevity of the dogs (Asher et al., 2009; Careau et al., 2010; O'neill et al., 2013; Summers et al., 2010; Urfer et al., 2020). Owing to the aging of dogs and the consequent increase in disorders with significant health costs, it is crucial to encourage aging research concerning biological effects as an approach to enhancing healthy longevity with substantially improved quality of life. Although these biological assessments are well‐established and accepted in human medicine, there is a scarcity of correlating data in veterinary medicine (Lee et al., 2020).

Studies indicate that aging affects the physiology of clinically healthy elderly dogs, leading to changes in their serum biochemical profiles and haematological profile (Radakovich et al., 2017). Studies report that these haematological and biochemical indicators offer more objective insights into their physiological state compared to the physical examination (Metzger & Rebar, 2012). Additionally, as dogs age, their immune system experiences a gradual decline in function known as immunosenescence (Day, 2010; Holder et al., 2018; Rosato & Salsano, 2008). Many aspects of the canine immune system remain unknown in older dogs, including the innate immune response and the role of inflammation in various illnesses. Understanding the immune system's functional capacity at different life stages could assist veterinarians in developing preventive and therapeutic strategies to improve healthy aging and overall longevity in dogs (Pereira et al., 2019).

Healthy aging can be promoted by using a supplement‐based therapeutic approach having potent adaptogenic, haemopoietic and antioxidant properties. One such important plant is the *Withania somnifera* (L.) *Dunal* (ashwagandha), which is an extremely valuable plant that influences key physiological processes, including the neurological, immunological, endocrine and reproductive systems (Mukherjee et al., 2021). It is widely recognized for its ability to attenuate stress responses in both humans as well as animals (Kulkarni & Dhir, 2008). Several studies involving humans have shown that *W. somnifera* reduces stress (Choudhary et al., 2017; Salve et al., 2019). In a study conducted on geriatric canines with liver dysfunction, ashwagandha root extract (ARE) supplementation illustrated hepatoprotective, antioxidant and anti‐peroxidative effects (Nabi et al., 2014). Withanolides like withaferin‐A are the primary active constituents behind the pharmacological effects of ARE. Withaferin has been proven as a strong immunomodulator, leptin sensitizer, antioxidant, anti‐diabetic, hepatoprotective and anti‐pancreatitis agent (Gu et al., 2020; Jadeja et al., 2015; Lee et al., 2016; Peddakkulappagari et al., 2019; Tekula et al., 2018; Tiruveedi et al., 2018). However, clinical studies assessing stress mitigating and immunomodulatory effects of ARE and withanolides in healthy geriatric dogs are rather limited.

Based on the available evidence, we hypothesized that ARE can reduce the effects of advancing age like physiological alterations, dysfunctional immune system and susceptibility to diseases by its immunomodulatory effects. The aim of this study was to study the effects of a standardized *W. somnifera* root extract rich in withanolides on stress (cortisol), haematological (RBC, WBC and haemoglobin), biochemical (liver and kidney function biomarkers), antioxidant (antioxidant enzymes and lipid peroxidation) and anti‐inflammatory parameters (cytokines and transcription factors) in healthy geriatric dogs.

## 2 MATERIALS AND METHODS

### 2.1 Materials

KSM‐66 high concentration ARE was provided as a gift sample by Ixoreal BioMed Inc.. Acetic acid (Cat. No. 11040), 5,5′‐dithio‐bis‐(2‐nitrobenzoic acid) (Cat. No. D8130), sodium dodecyl sulphate (Cat. No. 32096), potassium chloride (Cat. No. 39594 K05), sodium hydroxide (Cat. No. 41067 K05), hydrochloric acid (Cat. No. 320331) and sodium chloride (Cat. No. S0171) were purchased from Sigma Aldrich. Glutathione reduced (Cat. No. 074011) was purchased from Sisco Research Laboratories Pvt. Ltd. 2‐Thiobarbituric acid (TBA) (Cat. No. RM1594) and dipotassium hydrogen phosphate (K_2_HPO_4_) (Cat. No. RM1045) were procured from Himedia Lab. Pvt. Ltd. All the other chemicals were of research grade unless otherwise stated. The enzyme linked immunosorbent assay (ELISA) kits for cortisol (Cat. No. CK‐bio‐18938), IFN‐γ (Cat. No. CK‐bio‐18858), TNF‐α (Cat. No. CK‐bio‐18955), IL‐10 (Cat. No. CK‐bio‐18860), Nrf‐2 (Cat. No. CK‐bio‐20668) and NFκB (Cat. No. CK‐bio‐20649) were procured from Shanghai Coon Koon Biotech Co., Ltd.

### 2.2 Study design

The randomized, double‐blind, placebo‐controlled trial was conducted from July 2022 to September 2022 at a Pet clinic in Telangana, India. The study protocol was approved by the Institutional Animal Ethics Committee and the Committee for Control and Supervision of Experiments on Animals (CCSEA), New Delhi, India. The animals were humanely treated and any discomfort was prevented by employing appropriate care and handling procedures with minimal stress and clinical supervision by an expert veterinarian.

### 2.3 Study animals, randomization and intervention

A total of 20 visibly healthy, obese geriatric Labrador dogs aged ≥8 years with normal haemato‐biochemical parameters were enrolled, irrespective of their gender. All the dogs enrolled were client‐owned, living in Telangana, India, and were recruited at the Pet clinic during routine visits. Informed consent was signed by the owners of the dogs at the screening visit prior to initiation of the study. Dogs who were sick, anaemic, <6 years, and with dermatological conditions, neuromuscular disorders, parasitic worm load and abnormal haemato‐biochemical parameters were excluded.

**FIGURE 1:**
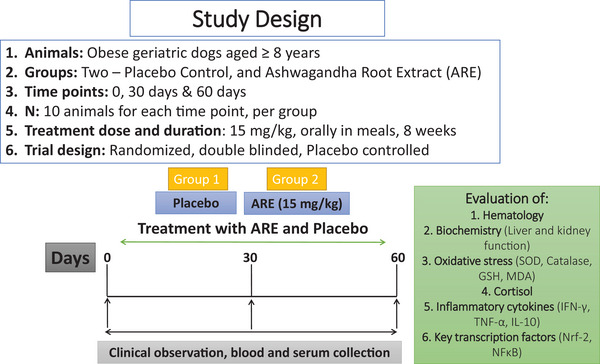
The pictorial representation of the study design. IFN‐γ, interferon‐γ; Nrf‐2, nuclear factor erythroid 2‐related factor 2.

The enrolled dogs were randomly assigned to 1 of the 2 groups (placebo and ARE) in a 1:1 randomization ratio. Thus, 10 dogs were in the ARE group, and another 10 dogs were in the placebo group (Figure 1). Randomization was performed using an automated method of random number generation which was pre‐determined for the site. The investigational product and the placebo group products were manufactured and packed into identical containers and labelled equivalently to ensure blinding. All dogs were assessed at baseline, on day 30, and day 60.

### 2.4 Study visits

A veterinarian examined each dog and interviewed the owner at the baseline and end of the study. At the baseline (day 0), a physical examination and eligibility evaluation based on the inclusion and exclusion criteria and psychometric scales were done, followed by blood sampling and dispensing of trial intervention to the dog owner with instructions for daily administration. At the end of the study visit (day 60), physical examination, blood sampling, psychometric scales and adverse events evaluation were performed.

### 2.5 Trial interventions

The treatment intervention contained a commercial ARE (KSM‐66 ARE), obtained as a gift sample from the manufacturer, Ixoreal Biomed Inc. The product contains more than 5% of total withanolides and the extract is prepared via a green chemistry‐based process. The placebo group received starch, which was packed identically to the investigational product as well as was identical in appearance, colour, odour and taste. The selected dogs received either ARE or placebo powder orally, once daily by mixing in the dinner meal at a dose of 15 mg/kg body weight. The dog owners were instructed to give the powder as per animal body weight by uniformly mixing in the regular meal. The dose was selected based on earlier published literature (Kaur et al., 2022).

### 2.6 Blood sample collection

The animal blood collection was carried out at baseline, day 30 and day 60 to analyse different parameters. The blood samples were collected from the cephalic vein. The dogs were restrained manually, before sample collection, by a veterinarian. A non‐slippery mat was used to hold the dog steady during sample collection. All the dogs were acclimatized to restraint and the sound of the clippers before initiation of the study. The collection site was shaved with an electric clipper. The site was cleaned with three alternating scrubs of 70% isopropyl alcohol and betadine disinfectant. Blood was collected from the dog's forelimb, starting distally (near the paw) and working proximally (away from paw) with a 21‐gauge needle. Separate plastic vacutainers containing a clot activator were used to collect the blood for serum samples. After sample collection, the blood flow was stopped by applying pressure with sterile gauze to the sampling site for approximately 30 s to achieve haemostasis. Serum samples were separated from the blood by using centrifugation at 4000 rpm for 15 min. The evaluation of serum biochemical parameters was followed thereafter.

### 2.7 Estimation of haematological parameters

ARE is a known adaptogen and may improve the overall haematological profile of aging animals. To this end, total erythrocyte count, total leucocyte count and haemoglobin were assessed from the blood samples of the dogs using a haematology analyser (DH36 VET, Dymind Biotech). Total erythrocyte count was measured in million cells/mm^3^ and total leucocyte count was measured in ×10^9^ cells per litre (×10^9^ cells/L), whereas haemoglobin was expressed in grams per decilitre (g/dL).

### 2.8 Serum biochemical parameters

To measure the effects of intervention on liver and kidney function, we measured some of the relevant biomarkers (Devarasetti et al., 2024). For liver function, measurements of serum alanine aminotransferase (ALT), aspartate aminotransferase (AST), alkaline phosphatase (ALP), total protein, albumin and globulin were performed. On the other side, creatinine and blood urea nitrogen (BUN) were measured for the estimation of effects on kidney function. The analysis was performed using commercially manufactured kits. ALT, AST and ALP were expressed in international units per litre (IU/L), total protein, albumin and globulin were expressed in grams per decilitre (g/dL), whereas creatinine and BUN were expressed in milligrams per decilitre (mg/dL).

### 2.9 Measurement of antioxidant parameters in serum

Oxidative stress is an important hallmark of age related general physiological decline. Antioxidant markers, such as SOD, catalase, GSH and MDA, were evaluated to understand the effect of interventions on oxidative stress. The measurements of SOD and catalase were conducted as per an earlier method (Khurana et al., 2019; Madesh & Balasubramanian, 1998). The measurement of GSH was conducted by using Ellman's reagent and MDA was evaluated by the method based on TBA reactive substances (De Leon & Borges, 2020; Khurana et al., 2019; Rahman et al., 2006). Reduced SOD was expressed in units per milligram (U/mg) of protein, catalase was in nanomoles per minute per millilitre (nmol/min/mL), GSH in milligram per decilitre (mg/dL) and MDA in nanomoles per millilitre (nmoles/mL).

### 2.10 Measurement of serum cortisol levels

The ELISA‐based commercial kit was used to measure cortisol levels in the serum. The procedure involved the use of a precoated plate, followed by incubation with the samples and washing with wash buffer. The samples were then incubated with HRP linked secondary antibody followed by incubation with chromogen. The reaction was terminated by the use of a stop solution. The colorimetric signal was evaluated at 450 nm optical density and compared to the standard curve. The concentration of cortisol in the serum was measured in nanograms per millilitre (ng/mL).

### 2.11 Anti‐inflammatory parameters

The anti‐inflammatory parameters, including IL‐10, INF‐γ, NFκB, Nrf‐2 and TNF‐α, were assessed using ELISA and were expressed in picogram per millilitre (pg/mL). Briefly, the samples (50 µL) were incubated in the respective precoated plate for 1 h at 37°C, followed by washing with wash buffer. Next, the samples were incubated with HRP linked antibody for 1 h at 37°C followed by washing with wash buffer. The samples were then incubated with chromogen solution A and B for 15 min at 37°C in darkness. The reaction was stopped by the addition of stop solution followed by measurement of optical density at 450 nm. The quantification of sample absorbance was performed by using a standard curve.

### 2.12 Statistical analysis

A descriptive statistical analysis was performed. Data were presented as mean ± SD. All the efficacy and safety between the groups were assessed using two‐way ANOVA followed by Bonferroni's Posthoc analysis. To analyse the efficacy within the groups, *p* < 0.05 was considered statistically significant. Graphpad Prism version 5.0 was used for statistical analysis (Patel et al., 2023).

## 3 RESULTS

### 3.1 Effect of ARE intervention on haematological parameters

**FIGURE 2:**
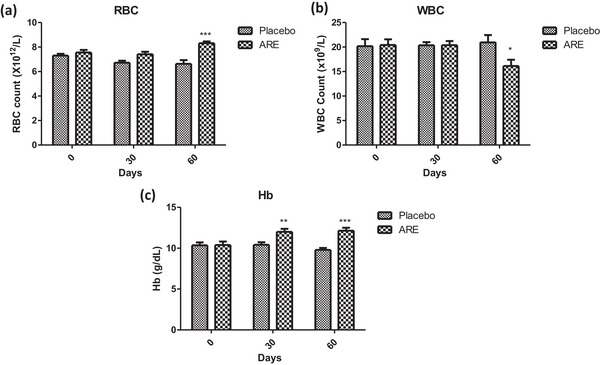
Effect of treatment interventions on haematological parameters: (a) red blood cell count (RBC); (b) white blood cell count (WBC); (c) haemoglobin (Hb). Data *n* = 10; statistically analysed by mean ± SD. *Significantly different from placebo group at *p* < 0.05, **Significantly different from placebo group at *p* < 0.01 and ***significantly different from placebo group at *p* < 0.001. ARE, ashwagandha root extract.

Experimentally, all the 20 dogs completed the entire study without any health issues and exclusion. The mean age of dogs in ARE and placebo groups was 10.04 ± 1.10 and 14.79 ± 2.81 years, respectively. A haematological profile is an important indicator of the overall health of an animal. The mean concentrations of haematological parameters were assessed at baseline, day 30, and day 60 in the ARE and placebo‐treated groups. At the end of 60 days, erythrocyte and haemoglobin count increased significantly in the ARE‐treated dogs to 8.31 ± 0.41 × 10^12^ cells/mL and 12.15 ± 1 g/dL compared to 7.55 ± 0.6 cells/mL and 10.39 ± 1.21 g/dL, respectively, compared to the placebo (*p* < 0.001, Figure 2a,c, Table 1). The values were 6.63 ± 0.89 × 10^12^ cells/L (RBCs) and 9.79 ± 0.70 g/dL (Hb), in case of placebo group at the end of 60 days, showing a significant difference compared to the ARE group (Figure 2a,c, Table 1). On the other side, leukocyte count significantly decreased from a baseline value of 20.42 ± 3.31 × 10^9^ cells/L to 16.14 ± 3.60 × 10^9^ cells/L at day 60 (*p* < 0.05, Figure 2b, Table 1), indicating that ARE potentially reduced the overall inflammatory state of the animals, improving their overall wellbeing. However, the changes remained within the reference intervals throughout the study. These results show that at day 60, the mean values of the total erythrocyte count (*p* < 0.001) and Hb (*p* < 0.001) were significantly higher in ARE‐treated dogs, whereas leucocyte count was significantly lower (*p* = 0.007) compared to placebo (Figure 2 and Table 1).

**TABLE 1:** The effect of different treatment interventions on selected haematological parameters at different time intervals.

Groups	Baseline mean (SD)	Day 30 mean (SD)	Day 60 mean (SD)	*p*‐values^*^ (between baseline and day 60)	*p*‐value^*^ (between group)
**Erythrocyte count (10^12^/L)**					
ARE	7.55 (0.60)	7.41 (0.58)	8.31 (0.41)	<0.001	<0.001
Placebo	7.31 (0.41)	6.72 (0.47)	6.63 (0.89)	0.013	
**Leucocyte count (10^9^/L)**					
ARE	20.42 (3.31)	20.39 (2.36)	16.14 (3.60)	0.05	0.007
Placebo	20.17 (4.17)	20.36 (1.86)	20.95 (4.32)	0.226	
**Haemoglobin (g/dL)**					
ARE	10.39 (1.21)	12.01 (1.01)	12.15 (1.00)	<0.001	<0.001
Placebo	10.35 (1.03)	10.42 (0.91)	9.79 (0.70)	0.066	

Abbreviation: ARE, ashwagandha root extract.

* Statistically significant. *p* < 0.05 is considered statistically significant.

### 3.2 Effect of ashwagandha intervention on biochemical parameters of liver health

**FIGURE 3:**
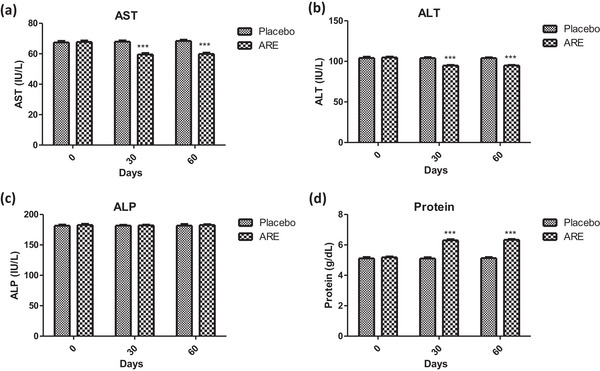
Effect of treatment interventions on the liver function markers: (a) aspartate aminotransferase (AST); (b) alanine aminotransferase (ALT); (c) alkaline phosphatase (ALP); (d) protein. Data *n* = 10; statistically analysed by mean ± SD. ***Significantly different from placebo group at *p* < 0.001. ARE, ashwagandha root extract.

**FIGURE 4:**
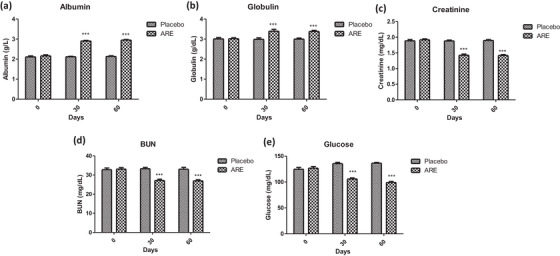
Effect of treatment interventions on important serum markers: (a) albumin; (b) globulin; (c) creatinine; (d) blood urea nitrogen (BUN); (e) glucose. Data *n* = 10; statistically analysed by mean ± SD. ***Significantly different from placebo group at *p* < 0.001. ARE, ashwagandha root extract.

Figures 3 and 4 depict the graphical representation of the biochemical parameters assessed at baseline, day 30, and day 60. For the measurement of liver health, the levels of AST, ALT, ALP, protein, albumin and globulin were used, whereas creatinine and blood urea nitrogen levels were evaluated to measure the effects of ARE on kidney function. The levels of AST and ALT were significantly lower in ARE‐treated dogs at day 30 (*p* < 0.001, Figure 3a) and day 60 (*p* < 0.001, Figure 3b) compared to the placebo treated group. However, there was no significant difference in the levels of ALP among groups throughout the study duration (Figure 3c). Intervention with ARE led to significant improvement in the levels of serum proteins at day 30 (*p* < 0.001, Figure 3d) and day 60 (*p* < 0.001, Figure 3d) compared to the placebo treated group. The levels of serum albumin and globulin also improved significantly at day 30 (*p* < 0.001 for both, Figure 4a,b) and day 60 (*p* < 0.001 for both, Figure 4a,b) compared to the placebo treated animals. However, all the changes were within the reference ranges throughout the study.

The levels of kidney function markers also showed an interesting trend in the ARE‐treated group. The levels of serum creatinine were significantly reduced at day 30 (*p* < 0.001, Figure 4c) and day 60 (*p* < 0.001, Figure 4c) in the ARE‐treated dogs compared to the placebo group. Similarly, the levels of BUN were observed to be significantly reduced at day 30 (*p* < 0.001, Figure 4d) and day 60 (*p* < 0.001, Figure 4d) in the ARE‐treated dogs compared to the placebo group, indicating that ARE improved the overall kidney function by virtue of its adaptogenic effects. Interestingly, the serum levels of glucose were also reduced in the ARE‐treated animals at both the time intervals (*p* < 0.001, Figure 4e) compared to the placebo group. The values of glucose were within the standard range; however, these results support the well‐known glucose lowering effects of the ashwagandha withanolides.

### 3.3 ARE extract reduces oxidative stress in geriatric dogs

**FIGURE 5:**
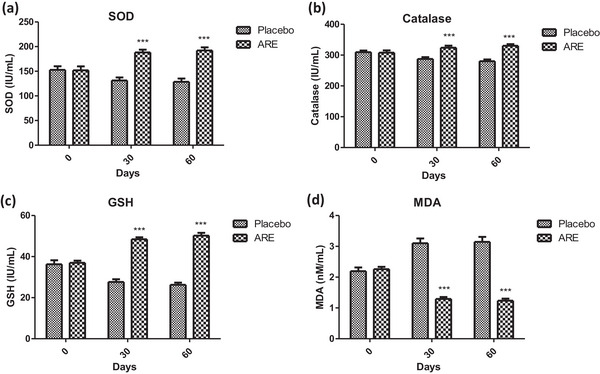
Effect of treatment interventions on the markers of oxidative stress: (a) superoxide dismutase (superoxide dismutase [SOD]); (b) catalase; (c) glutathione (GSH); (d) malondialdehyde (MDA). Data *n* = 10; statistically analysed by mean ± SD. ***Significantly different from placebo group at *p* < 0.001. ARE, ashwagandha root extract.

Oxidative stress is a key process driving cellular senescence and its reversal is known to improve overall cellular health and longevity. In this study, the levels of four key oxidative stress markers, that is SOD, catalase, GSH and MDA, were recorded at different study intervals during the treatment regimen. SOD is an important redox enzyme and its levels were significantly increased in the ARE‐treated group at day 30 (*p* < 0.001, Figure 5a) and day 60 (*p* < 0.001, Figure 5a, Table 2) when compared to the placebo treated group. Catalase is another critical enzyme required for redox homeostasis. The levels of catalase were found to be significantly increased in the ARE treated dogs when compared to the placebo group at both day 30 (*p* < 0.001, Figure 5b, Table 2) and day 60 (*p* < 0.001, Figure 5b, Table 2). Glutathione is an important tripeptide that plays a vital role in cellular antioxidant defence. The levels of GSH were found to be significantly enhanced in the ARE‐treated group at day 30 (*p* < 0.001, Figure 5c, Table 2) and day 60 (*p* < 0.001, Figure 5c, Table 2) when compared to the placebo group. Lipid peroxidation is an oxidative stress associated problem associated with oxidation of membrane proteins. MDA levels were assessed to measure the extent of lipid peroxidation by the TBARS assay. The levels of MDA were found to be significantly reduced in the ARE‐treated groups when compared to the placebo group at both day 30 (*p* < 0.001, Figure 5d, Table 2) and day 30 (*p* < 0.001, Figure 5d, Table 2), clearly indicting the strong antioxidant effects of ARE.

**TABLE 2:** The effect of different interventions on selected antioxidant parameters at different time intervals.

Groups	Baseline mean (SD)	Day 30 mean (SD)	Day 60 mean (SD)	*p*‐values^*^ (between baseline and day 60)	*p*‐value^*^ (between‐group)
**SOD (IU/mL) (range: 170–200 U/mL)**					
ARE	151.99 (22.39)	188.33 (15.60)	192.18 (18.36)	<0.001	<0.001
Placebo	152.84 (20.58)	131.28 (17.76)	128.64 (18.88)	0.027	
**Catalase (IU/mL)**					
ARE	307.91 (20.92)	323.76 (19.29)	330.22 (15.56)	<0.001	<0.001
Placebo	309.57 (15.59)	287.49 (16.83)	280.00 (16.56)	<0.001	
**GSH (IU/mL) (range: 40–50 IU/mL)**					
ARE	36.96 (2.99)	48.44 (2.66)	50.22 (3.85)	<0.001	<0.001
Placebo	36.29 (5.52)	27.69 (3.67)	26.27 (3.05)	<0.001	
**Malondialdehyde (nM/mL) (range: 01–02 nM/mL)**					
ARE	2.26 (0.22)	1.29 (0.19)	1.24 (0.19)	<0.001	<0.001
Placebo	2.20 (0.34)	3.10 (0.44)	3.15 (0.45)	<0.001	

Abbreviations: ARE, ashwagandha root extract; GSH, glutathione; SOD, superoxide dismutase.

* statistically significant. *p* < 0.05 is considered statistically significant.

### 3.4 Effect of ARE intervention on cortisol levels

**FIGURE 6:**
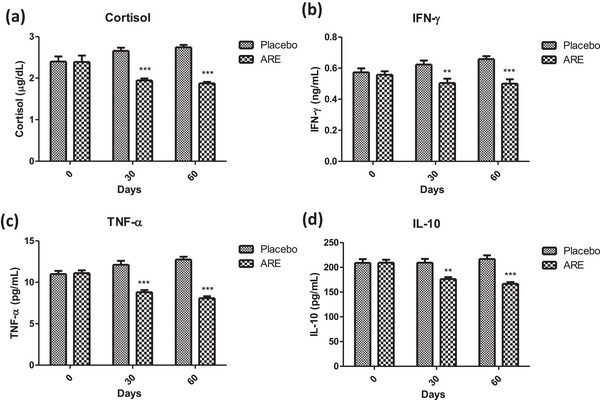
Effect of treatment interventions on cortisol and cytokines: (a) cortisol; (b) interferon‐γ (IFN‐γ); (c) tumour necrosis factor‐α (TNF‐α); (d) interleukin‐10 (IL‐10). Data *n* = 10; statistically analysed by mean ± SD. **Significantly different from placebo group at *p* < 0.01, ***significantly different from placebo group at *p* < 0.001. ARE, ashwagandha root extract.

Cortisol is a stress response hormone and its levels correlate with the age and the general wellbeing of the animals. In this study, the levels of serum cortisol were assessed at baseline, day 30, and day 30 to ascertain the effect of ARE. It was observed that the serum cortisol level in the placebo group increased from baseline value of 2.4 to 2.75 µg/dL at day 60 (*p* < 0.001, Figure 6a), whereas it reduced significantly in the ARE treated group to 1.88 µg/dL at day 60 (*p* < 0.001). On the other side, there was a trend in increase in cortisol levels in the placebo control group over the study duration. Further, the difference in serum cortisol levels between both groups at days 30 and 60 was statistically significant (*p* < 0.001 at both intervals, Figure 6a).

### 3.5 Effect of ARE intervention as an anti‐inflammatory agent

Progression of age is characterized by a generalized state of overall increased basal inflammatory state. To this end, we probed the effect of ARE intervention in geriatric dogs by measurement of two important proinflammatory cytokines (IFN‐γ, TNF‐α) and one anti‐inflammatory cytokine (IL‐10). The levels of IFN‐γ showed a trend of increase in the placebo control group over the days 30 and 60 period, though the changes were non‐significant (Figure 6b). Interestingly, the levels of IFN‐γ were significantly lower at day 30 (*p* < 0.01, Figure 6b) and day 60 (*p* < 0.001, Figure 6b) in the ARE‐treated group when compared to the placebo group. A similar trend was observed in the case of TNF‐α with significant reduction in the ARE treated group at day 30 (*p* < 0.001, Figure 6c) and day 60 (*p* < 0.001, Figure 6c) compared to the placebo control group. On the other side, IL‐10 is a known anti‐inflammatory cytokine. As the overall inflammatory state was reduced in the ARE‐treated dogs, the levels of IL‐10 showed reduction on the day 30 (*p* < 0.01, Figure 6d) and day 60 (*p* < 0.001, Figure 6d) time points when compared to the placebo control group. These results indicate the strong anti‐inflammatory effects of ARE, which may be of potential clinical relevance for reducing age associated inflammation in dogs.

### 3.6 ARE intervention showed anti‐inflammatory effects by modulation of Nrf‐2 and NFκB

**FIGURE 7:**
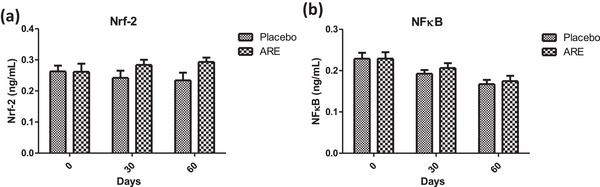
Effect of treatment intervention on Nrf‐2 and NFκB: (a) Nrf‐2 and (b) NFκB. Data *n* = 10; statistically analysed by mean ± SEM. ARE, ashwagandha root extract; NFκB, nuclear factor kappa light chain enhancer of activated B cells; Nrf‐2, nuclear factor erythroid 2‐related factor 2.

The interesting effects of ARE on the measured cytokine profile motivated us to probe the potential mechanism behind the observed effects. Nrf‐2 and NFκB are two key transcription factors which regulate multiple genes associated with the synthesis of cytokines and oxidative stress related proteins and enzymes. There was a trend towards an increase of Nrf‐2 throughout the study duration in the ARE‐treated group compared to the placebo control, though the levels were non‐significant (*p* < 0.293) among groups (Figure 7a). On the other side, the levels of NFκB showed a trend of decline in the ARE treated group over the intervention period when compared to the placebo treated group (Figure 7b). However, the results were non‐significant (*p* < 0.293).

## 4 DISCUSSION

Clinically healthy, elderly dogs of various breeds living in diverse habitats and eating different foods can potentially exhibit differential physiological profiles (Fahey et al., 2008). These changes might be visible in the form of changes in haematological profile and vital organ functions like that of the kidney and the liver (Lee et al., 2020). Further, the oxidative stress and inflammatory state of the animals might be significantly different with significantly declining trends when compared to young adults (Wallis et al., 2020; Watowich et al., 2020). This reflects the modified physiology or pathology in geriatric dogs vs. their younger counterparts (Day, 2010). Haematological and biochemical indicators are typically more objective and specific than physical exams for the assessment of physiological state. The current study investigated the adaptogenic property of orally administered ARE at 15 mg/kg in a double‐blind, placebo‐controlled trial in geriatric dogs by evaluation of important physiological parameters, including haematological profile, biochemical markers, antioxidant indicators, serum cortisol levels and anti‐inflammatory responses. The study provides new dimension to the adaptogenic potential of ARE in geriatric dogs. Oral administration of ARE exhibited promising results in managing age‐related changes in geriatric dogs without any adverse events.

The health of blood has direct correlation with the overall wellbeing of an animal including the parameters like RBC and WBC count as well as Hb. Higher RBC and Hb can be markers of the quality of oxygenation, an important physiological feature required to maintain healthy functioning (Harper et al., 2003; Lowseth et al., 1990). The beneficial effects of ARE on erythrocyte count and Hb levels, coupled with a decrease in leukocyte count within reference intervals, are consistent with previous findings. Raut et al. (2012) conducted a study that demonstrated an increase in red blood cell indices with ashwagandha (750–1250 mg/kg), which could be attributed to its potential haematopoietic effects. The decrease in leukocyte count may indicate that ARE does not lead to immunosuppression but rather maintains white blood cell counts within a healthy range. It also indicates that steady state inflammation is substantially reduced in geriatric dogs, an effect which is very beneficial.

Aging is well known to affect vital organ functions, which culminate in the form of perturbed serum levels of biomarkers of liver and kidney function (Alexander et al., 2018; Miyagawa et al., 2010). The effect of ARE on biochemical parameters, including ALT, AST, glucose, creatinine, BUN, protein, albumin and globulin levels, indicates potential benefits for liver function and metabolic regulation. The decrease in ALT and AST levels as well as improvement of liver derived proteins, that is, albumin and globulin, suggest improved liver health. Further, increases in protein, albumin and globulin levels are consistent with a positive impact on nutritional status and demonstrate ARE's role in the improvement of immune responses, which is crucial in managing physiological changes. On the other side, changes in glucose and kidney function markers (creatinine and BUN) may be indicative of overall metabolic improvements. These findings align with previous studies that have highlighted ashwagandha's hepatoprotective effects in humans (Verma et al., 2021).

Oxidative stress is one of the most important physiological processes that initiates, maintains and drives cellular senescence. Geriatric dogs show an increased basal oxidative stress (Alexander et al., 2018; Ferreira et al., 2014). Herein, a significant increase in the levels of SOD, catalase and GSH was observed with ARE intervention at days 30 and 60 compared to the placebo control group. SOD is a crucial enzyme in the antioxidant defence system that plays a key role in detoxifying superoxide radicals. This increase in SOD levels suggests that ashwagandha supplementation enhances the body's ability to combat oxidative stress. However, catalase is an enzyme responsible for breaking down hydrogen peroxide, a harmful reactive oxygen species (Todorova et al., 2005). The increase in catalase activity suggests that ARE may contribute to reducing oxidative damage by promoting the breakdown of hydrogen peroxide. Previous studies have shown the beneficial effect of ashwagandha on SOD and catalase (Ahmad et al., 2010). Furthermore, GSH is a key intracellular antioxidant, and its increase may indicate an enhanced cellular defence against oxidative stress (Lu, 2013). On the other side, MDA is a marker of lipid peroxidation and oxidative stress. The significant decrease in MDA levels suggests that ARE may effectively reduce oxidative damage to lipids. Several studies have supported this finding, demonstrating ashwagandha's ability to reduce lipid peroxidation by quenching free radicals and enhancing antioxidant defences (Khalil et al., 2023). The observed improvements in the levels of oxidative stress markers might be due to the known antioxidant effects of withaferin‐A, a well‐known withanolide found in ashwagandha extracts (Tekula et al., 2018; Tiruveedi et al., 2018).

Cortisol levels are indicative of a state of stress and tend to increase in the aging population. Increased cortisol levels mean that the overall aging process is heightened, the immune response might be declining and the overall wellbeing of the animal is compromised (Mongillo et al., 2014). The significant reduction in serum cortisol levels in the ARE group compared to an increase in the placebo group reflects the anti‐stress properties of ashwagandha. This aligns with several existing studies that have investigated the cortisol‐modulating effects of ashwagandha. Kaur et al. (2022) studied the effect of ashwagandha on reducing stress and anxiety in domestic dogs aged 3–8 years and reported that ashwagandha was associated with significant reductions in urine cortisol–creatinine ratio, signs of fear and anxiety and pain interference. Priyanka et al. (2020) also reported a reduction in cortisol levels in horses with ashwagandha. Similar findings are also evident in human studies (Salve et al., 2019).

Inflammation is an important indicator of physiological response to wear and tear as well as defence against invaders. Aging tends to correlate with declining immune response culminating in the form of an elevated basal inflammatory state (Greeley et al., 1996). The significant decrease in pro‐inflammatory cytokines (IFN‐γ, TNF‐α and NFκB) and anti‐inflammatory cytokine (IL‐10) levels observed with ARE supplementation is promising for its potential anti‐inflammatory effects. This is in line with its immunomodulatory effects documented in the literature. Misra et al. reported a reduction in TNF‐α levels, aligning with the anti‐inflammatory effects observed in this study (Kumar et al., 2022). Additionally, Khan et al. (2006) investigated ashwagandha's immunomodulatory effects in animals and found that it reduced pro‐inflammatory cytokines while increasing anti‐inflammatory cytokines, further supporting the immunomodulatory properties of Ashwagandha. In addition, a modulatory trend in the levels of Nrf‐2 and NFκB was observed with ARE intervention, two of the key transcription factors regulating the physiological oxidative and inflammatory state. These findings collectively highlight ashwagandha's potential to modulate the immune response by regulating cytokine levels, which may have therapeutic implications in other veterinary chronic inflammatory conditions like rheumatoid arthritis, psoriasis, mastitis and others.

To the best of our knowledge, this is the first ever study to explore the potential of ARE to mitigate these age‐related changes in healthy geriatric dogs. This study's comprehensive assessment of a wide range of parameters, including haematological profiles, biochemical markers, antioxidant indicators, cortisol levels and anti‐inflammatory responses, offers a holistic perspective on the effects of ashwagandha. Furthermore, the longitudinal analysis with multiple assessment points over a 2‐month period allows for a thorough examination of changes over time. Another dimension of the clinical application of ARE is the withanolides content in the extract; the higher the content, the superior the activity of the extract. Poor pharmacokinetics is one of the important bottlenecks in the clinical translation of herbal medicines from laboratory studies (Sayed et al., 2019). Withaferin A is one of the widely studied withanolides with a known poor bioavailability (Gupta et al., 2022). Achieving higher withanolide content in the extract is a desirable trait for a high‐quality extract to elicit immunomodulatory benefits.

The main limitation of the study was the use of asingle dog breed and a small sample size. In future, further studies with a larger sample size and specification regarding the diversity of dog breeds would provide more insight into how the results apply to different breeds with varying susceptibilities to age‐related changes. To achieve wide scale clinical translation for veterinary application, it is essential to perform more studies to understand the mechanism of action of ARE as well as studies targeted at safety profile of the extract. This will ensure that ARE can become widely available for its pharmacological effects based on sound scientific evidence.

## 5 CONCLUSION

In conclusion, this study provides valuable insights into the multi‐faceted effects of ashwagandha supplementation on various physiological parameters (hepatic and renal function markers, oxidative stress and cortisol) in aging dogs. These results indicate that ARE can be beneficial for enhancing the quality of life in aging canines; our results also prove that ARE can reduce the levels of cortisol in aging dogs, a marker of stress. These findings provide novel insights into the antioxidant and anti‐inflammatory effects of ARE (reduction of MDA, TNF‐α and IFN‐γ), supporting the potential use of ashwagandha in stress management, haematopoiesis, inflammation modulation, as well as oxidative stress reduction. This study has immense practical implications in clinical practice including the use of ashwagandha for reducing stress and improving the overall health; focusing on clinically healthy geriatric dogs, directly addressing a population often overlooked in research.

## AUTHOR CONTRIBUTIONS

**Kala Kumar Bharani**: Conceptualization; data curation; funding acquisition; methodology; project administration; resources; supervision; writing — review and editing. **Ashok Kumar Devarasetti**: Conceptualization; data curation; formal analysis; funding acquisition; methodology; project administration; resources; supervision writing — review and editing. **Latha Carey**: Formal analysis; investigation; methodology; supervision writing — review and editing. **Amit Khurana**: Conceptualization; formal analysis; methodology; supervision; validation; writing — original draft; writing — review and editing. **Rajesh Kollipaka**: Conceptualization; data curation; investigation; methodology; project administration; supervision; visualization; writing — review and editing. **Donga Durga Veera Hanuman and Anil Kumar Banothu**: Data curation; formal analysis; investigation; methodology; project administration; visualization; writing — review and editing. **Vinaya Sree Chetla**: Data curation; formal analysis; investigation; methodology; writing — review and editing.

## CONFLICT OF INTEREST STATEMENT

The authors declare that there are no conflicts of interest pertaining to the research described in this article.

## FUNDING INFORMATION

None.

### ETHICS STATEMENT

The study protocol was approved by the Institutional Animal Ethics Committee (IAEC) at the College of Veterinary Science, P.V.N.R. Telangana Veterinary University, Hyderabad (29/25/C.V.Sc, Hyderabad, dated 2/7/2022) and Committee for Control and Supervision of Experiments on Animals (CCSEA), New Delhi, India. The randomized, double‐blind, placebo‐controlled trial was conducted from July 2022 to September 2022 at Cure Pet Clinic, Hanamkonda, Telangana, India.

### PEER REVIEW

The peer review history for this article is available at https://publons.com/publon/10.1002/vms3.1556.

## Data Availability

The data will be made available upon reasonable request to the corresponding author.
